# Additive Manufacturing of Conducting Polymers: Recent Advances, Challenges, and Opportunities

**DOI:** 10.1021/acsapm.1c00252

**Published:** 2021-06-01

**Authors:** Miryam Criado-Gonzalez, Antonio Dominguez-Alfaro, Naroa Lopez-Larrea, Nuria Alegret, David Mecerreyes

**Affiliations:** † POLYMAT University of the Basque Country UPV/EHU, Avenida de Tolosa 72, 20018 Donostia-San Sebastián, Spain; ‡ Instituto de Ciencia y Tecnología de Polímeros CSIC, Juan de la Cierva 3, 28006 Madrid, Spain; § Ikerbasque, Basque Foundation for Science, 48013 Bilbao, Spain

**Keywords:** conducting polymers, additive manufacturing, 3D printing, PEDOT, electronic applications, inks, bioelectronics

## Abstract

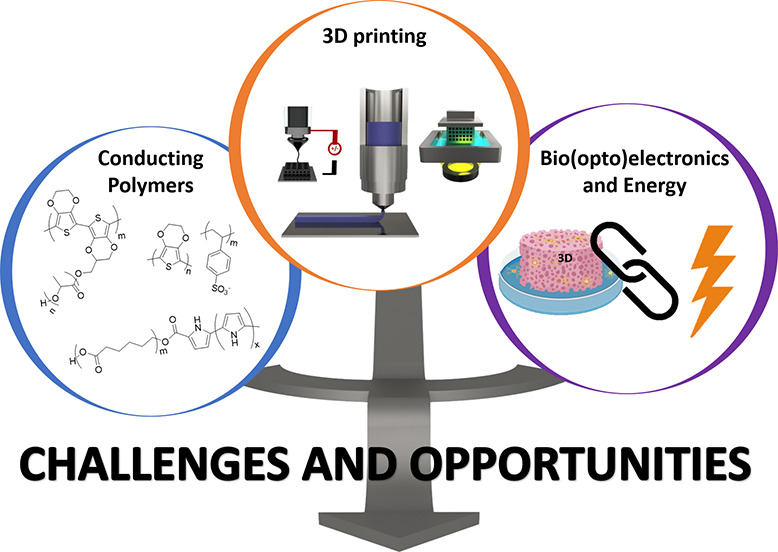

Conducting polymers (CPs) have been attracting great attention in the development of (bio)electronic devices. Most of the current devices are rigid two-dimensional systems and possess uncontrollable geometries and architectures that lead to poor mechanical properties presenting ion/electronic diffusion limitations. The goal of the article is to provide an overview about the additive manufacturing (AM) of conducting polymers, which is of paramount importance for the design of future wearable three-dimensional (3D) (bio)electronic devices. Among different 3D printing AM techniques, inkjet, extrusion, electrohydrodynamic, and light-based printing have been mainly used. This review article collects examples of 3D printing of conducting polymers such as poly(3,4-ethylene-dioxythiophene), polypyrrole, and polyaniline. It also shows examples of AM of these polymers combined with other polymers and/or conducting fillers such as carbon nanotubes, graphene, and silver nanowires. Afterward, the foremost applications of CPs processed by 3D printing techniques in the biomedical and energy fields, that is, wearable electronics, sensors, soft robotics for human motion, or health monitoring devices, among others, will be discussed.

## 1 Introduction

Conducting polymers (CPs), including poly(3,4-ethylene-dioxythiophene) (PEDOT), polypyrrole (PPy), and polyaniline (PANi), have been attracting increased interest for the development of several (bio)electronic and energy devices, that is, electrodes, biosensors, electronic skin, wearable electronics, human motion sensors, health monitoring, or soft robotics. Most of the current electronic devices are rigid and possess uncontrollable geometries and architectures that lead to poor mechanical properties presenting ion/electronic diffusion limitations.^1^ Therefore, the design of disruptive custom (bio)electronic devices is in the process of a transformation from traditional two-dimensional (2D) thin films to shape-conformable three-dimensional (3D) structures. Traditional manufacturing methods, including solvent casting or spin-coating, are not able to fulfill the third dimension requirement, being necessary the application of emerging additive manufacturing (AM) technologies to yield materials with a high spatial resolution.^2^ In this regard, different AM and 3D printing technologies have emerged in the last years as promising industrial manufacturing methods.^3^ Another important advantage of 3D printing technology is the possibility of fabricating multimaterial objects, comprising different materials, that is, metals, polymers, ceramics, etc., in different sections in only one printing process to fulfill specific requirements, that is, chemical, mechanical, thermal, electrical features, etc., of a wide application range.^4,5^ Regarding the electronic field, 3D printing represents a powerful tool for multifunctional electronic materials design and fabrication due to its excellent ability to customize complex, tunable, and low-cost three-dimensional structures at the micrometric scale.^6,7^

From the early stages, conducting (semi)conjugated polymers were known for their optoelectronic properties and unique electronic conductivity while having a polymeric nature. However, it is well-known that most CPs do not show the typical mechanical properties and easy processing of thermoplastic polymers such as polyethylene. In fact, most CPs are insoluble and infusible powdery materials difficult to process. For this reason, the extension of additive manufacturing methods to conducting polymers has been more difficult than to other polymer families. In this review, the adaptation of conducting polymers to the most commonly used AM and 3D printing techniques will be discussed. In addition to the CPs, 3D printing offers the possibility to use multifunctional inks and 3D structures whose properties can be tailored by incorporating specific polymers, nanofillers, ionic liquids (ILs), and other biological components, which confer to the final structure conductivity, an improvement of the mechanical and/or electrical properties, or biocompatibility. For this reason, this review will also cover the 3D printing of CPs reinforced with functional nanofillers such as carbon nanotubes (CNTs), graphene, and silver nanowires (Ag-NWs) that have been widely investigated in the last years.^8−10^ The synergistic effect between conducting polymers and conducting fillers allows an enlargement of the applicability of these materials.^9−12^

This review article provides an overview of the recent developments of conducting polymers for additive manufacturing technologies. First, we will shortly explain the different AM technologies used for the 3D printing of conducting polymer materials, that is, inkjet extrusion, electrohydrodynamic, and light-based printing. In each 3D printing method, we will describe examples of the most common CPs such as PEDOT, PPy, or PANi. This description will include examples of the additive manufacturing of different nanocomposites based on CPs and CNTs, graphene, or silver nanowires. In the final part of the review, the wide range of applications of the 3D printing of conducting polymers mostly in the biomedical field will be discussed. To conclude, the challenges and opportunities for the full development of additive manufacturing methods of conducting polymers will be highlighted.

## 2 Main Additive Manufacturing and 3D Printing Technologies Used for Conducting Polymers

3D printing is the manufacturing of a structure with a specific design using computer aided design (CAD) and computer aided manufacturing (CAM) software. Depending on the source, 3D printing methods can be classified as follows: (i) inkjet printing that uses controlled pulses for material deposition, (ii) extrusion-based printing, where the source can be considered a mechanical movement, (iii) electrohydrodynamic printing, which employs a controlled electric field for the deposition process, and (iv) light-based printing, where lasers or light-emitting diodes (LEDs) are used for the curing/printing process.^6,11^ This section collects the 3D printing processing of the most relevant conducting polymers nowadays.

### 2.1 Inkjet Printing

**Figure 1:**
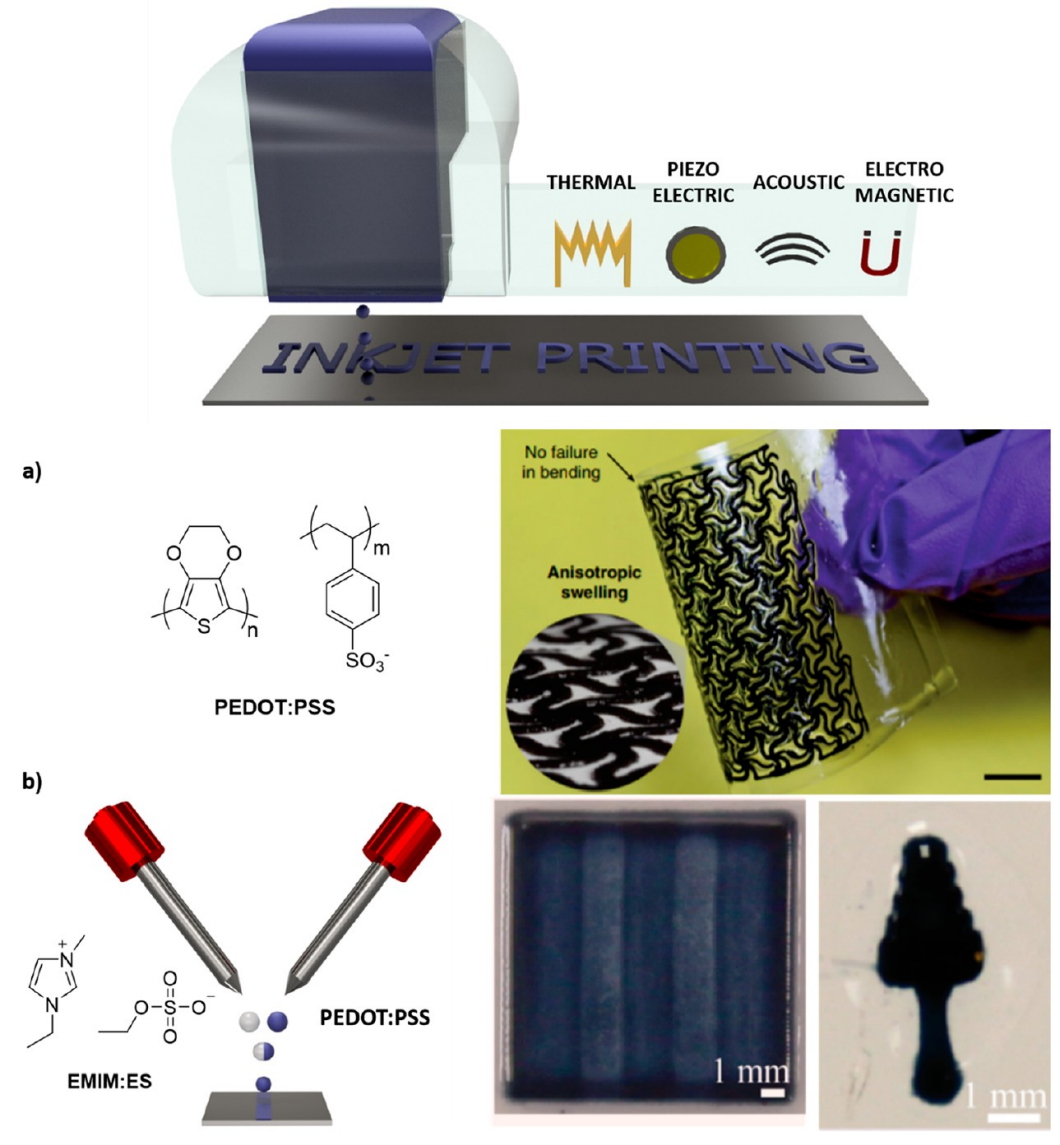
(top) General scheme of inkjet printing; the material with a low viscosity is deposited in drops due to a thermal, piezoelectric, acoustic, or electromagnetic input. (bottom) Two examples of PEDOT:PSS inks: (a) PEDOT:PSS/DMSO ink patterned on PET substrates forming complex structures. Adapted and reprinted with permission from ref (14). Copyright 2019 Springer Nature (b) Inkjet printing using coalescing pairs of PEDOT:PSS droplets with ionic liquids (ILs) to manufacture tridimensional structures. Adapted and reprinted with permission from ref (15). Copyright 2019 American Chemical Society.

Inkjet printing operates through the same mechanism as inkjet office printers, which means that material droplets are ejected from a cartridge due to the pressure generated from the formation and collapse of microbubbles inside the nozzle. The bubbles can be generated from a thermal, piezoelectric, or electromagnetic stimulus, and the material in the form of droplets is deposited on a surface (Figure 1).^12^ In contrast to extrusion-based printing, inkjet inks should be nonviscous to ensure the proper deposition and minimize the shear forces experienced by the material when it is ejected from the nozzle. Therefore, a viscosity lower than 100 mPa·s is recommended for inkjet inks.^13^ This opens the possibility of CPs to be formulated into solvent- or water-based inks for inkjet printing. However, the low solubility of CPs brings difficulties in the ink formulations.

#### Poly(3,4-ethylenedioxythiophene)

Nowadays, PEDOT is the most successful commercial CP in the (bio)electronics field due to its inherent properties, such as high conductivity, optical transparency in the form of thin films, and thermal and electrochemical stability.^16,17^ Moreover, PEDOT properties can be tuned through the use of counterions and secondary dopants as well as by polymer blending, processing, and post-treatment methods.^18−25^ The most successful commercial PEDOT material is an aqueous dispersion of poly(3,4-ethylenedioxythiophene) and poly(styrenesulfonate), named as PEDOT:PSS.^16,26^ The different processing methods of this PEDOT:PSS dispersion and the combination with other polymers and conducting fillers allow to tune the electrical, conducting, mechanical, and biological properties of the resulting materials.^27,28^

As a first example, the development of three-dimensional electrodes by the inkjet printing of PEDOT:PSS based materials will be shown.^29−31^ As a representative example, Bihar et al. reported the formulation of PEDOT:PSS in inks with a viscosity of 12.2 mPa·s and surface tension of 29 mN m^–1^ that were printed on a commercial stretchable polyamide textile (Dim, knee highs) leading to multilayer electrodes. The printing of PEDOT:PSS layers gave rise to a slight increase of rigidity, but electrodes could be stretched at least up to 200%. The resistance of the electrodes formed by 4, 6, 8, and 10 layers increased only by a factor of 6.0, 2.7, 1.4, and 1.5 times, respectively, at a 100% strain. Furthermore, the resistance of the electrodes consisting of eight printed PEDOT:PSS layers only increased by a factor of 3.5 when taken up to 200% strain.

In a second representative example, Zhao and co-workers prepared an ink based on PEDOT:PSS nanofibrils forming interconnected networks in a very simple method based on mixing volatile additive dimethyl sulfoxide (DMSO) into aqueous PEDOT:PSS solutions followed by a controlled dry-annealing and rehydration, forming hydrogels above 20 S cm^–1^. The ink was patterned on poly(ethylene terephthalate) (PET) substrates forming complex structures with a self-standing ability (Figure 1a).^14^ The inkjet technique has been also employed for printing coalescing pairs of PEDOT:PSS droplets simultaneously with ionic liquid (IL) 1-ethyl-3-methylimidazolium ethyl sulfate (EMIM:ES) droplets leading to an instantaneous gelation process to form highly conductive structures (Figure 1b). In this specific case, microreactive inkjet printing (MRIJP) was employed to pattern PEDOT:PSS/IL structures, with viscosities lower than 80 cP, by the in-air coalescence of PEDOT:PSS and IL droplets. PEDOT:PSS/IL films prepared by inkjet printing exhibited the same properties as those ones prepared by spin coating, with 89% of optical transmittance and electrical conductivity above 900 S cm^–1^. Moreover, it was demonstrated the possibility to deposit the 3D-conductive hydrogel through layer-by-layer to finally form patterned structures.^15^ Other ILs mixed with PEDOT:PSS were used to build stretchable devices that boosted the electronic conductivity up to 4100 S cm^–1^ under a 100% strain. Moreover, these inks were also explored for inkjet printing purposes forming complex structures used as interconnects for field-effect transistor arrays with a device density 5 times higher than the typical wavy metal interconnects.^32^

The incorporation of different conducting fillers, that is, carbon nanotubes (CNTs), graphene, and silver nanowires (Ag-NWs), into the conductive polymer matrices allows an enhancement of the final properties of the 3D printed materials as well as extending their applications.^33−38^ The employment of inkjet printing methods for the fabrication of conductive composite patterns of PEDOT:PSS incorporating multiwalled carbon nanotubes (MWCNTs) led to the orientation of the nanotubes in the printed sample with the subsequent electrical conductivity improvement. Samples with aligned MWCNTs showed a 53% enhanced conductivity in comparison with those ones randomly oriented. It was also observed that the orientation of the nanotubes into the ink was also controlled by their concentration, which means that, by increasing the MWCNTs from 0.01 to 0.05 wt %, percolated networks of well-distributed nanofillers in the printed samples could be obtained.^39^ Graphene has been also mixed with PEDOT:PSS to develop hybrid inks able to be processed by inkjet printing over a polyurethane support improving the thermoelectric properties.^40^ Furthermore, it was proven that graphene/PEDOT:PSS printed structures remained stable under static and dynamic bending (for 1000 cycles) conditions.^41^ The reinforcement effect of Ag-NWs in PEDOT:PSS-based materials has been also explored by inkjet printing. Multilayer films combining PEDOT:PSS and Ag-NWs layers exhibited good electrical properties reaching 10^2^ mA cm^–2^ by applying 1.5 V.^42^

#### Polypyrrole

The processing of PPy using additive manufacturing and 3D printing has been less explored than PEDOT. Ppy is a conducting polymer with a good biocompatibility and high electrical conductivity and is seen as an ideal candidate for applications in several fields, including chemical sensors and biomedical scaffolds.^37,43^ In an illustrative example, Weng et al.^44^ used inkjet printing technology to manufacture conductive polymer scaffolds by the interaction of PPy with different surfactants, to optimize the surface tension (30.8 mN m^–1^), viscosity (9.4 mPa s), and conductivity (1.26 S cm^–1^) of the inks, which were influenced by the oxidant concentration, and make them suitable for inkjet printing. The conductivity of the resulting printed films reached a value of 0.7 S cm^–1^. In another work, the same authors built up biocompatible scaffolds composed of PPy and collagen. For such a purpose, the PPy ink, mixed with ethanol as a cosolvent to decrease the surface tension and viscosity up to 9.39 cP, was printed over the polyarylate film with a customized waveform for 20 layers with the designed pattern reaching a conductivity of 1.1 S cm^–1^. Then, the collagen ink was jetted over the PPy lines for five layers, keeping constant the printing conditions in order to improve the cell adhesion properties of the 3D-printed scaffold.^45^

#### Polyaniline

PANi is a classical conducting polymer well-known by its different structures, electronic conductivity, and processability. The electroactive behavior of polyaniline is enhanced by doping with acids, whereas it is deteriorated by dedoping with bases. This doping/dedoping behavior of PANi, allowing to tune the electrical and electrochemical properties of this material,^46^ results in a poor stability.^47^ Bao et al.^48^ fabricated 3D hydrogel patterns by a sequential deposition of ammonium persulfate (1.05 cP) and a mixture formed by phytic acid and aniline (0.64–16.0 cP) employing inkjet printing and aerosol printing technologies. It is worth pointing out that the sequential deposition allowed a control of the viscosity, making the system suitable for inkjet printing. First, they printed the solution containing the oxidative initiator (ammonium persulfate) followed by printing the second solution containing the aniline monomer. Phytic acid played a double role, as it induced the gelation process and acted as a doping agent of PANi. The printed materials showed a highly hierarchical structure and a good electrical conductivity with a specific capacitance of ∼480 F g^–1^ and capacitance retentions of 91% and 83% over 5000 and 10 000 cycles, respectively. Rajzer and co-workers^49^ employed the inkjet printing technique to deposit a conductive PANi layer over a specific surface formed by osteoconductive materials (polycaprolactone, muskoskeletical disorders, gelatin, and calcium phosphate nanoparticles (SG5) obtaining 3D networks with a conductivity of ∼10^–3^ S cm^–1^. In that case, a suitable ink viscosity was obtained by centrifugation at specific conditions (4000 rpm, 30 min).

PANi can also act as a stabilizing agent of Ag-NWs to obtain hybrid inks with enhanced electrical properties as well as specific aspect ratios and viscosity (∼4.4 cP) that facilitate easy jetting and prevent clogging for optimal inkjet printing manufacturing. The Ag-NWs concentration was varied from 10 to 50 mg mL^–1^ in order to produce highly conductive patterns with a resistance lower than 50 Ω sq^–1^ in a minimal number of passes.^50^

### 2.2 Extrusion-Based Printing

**Figure 2:**
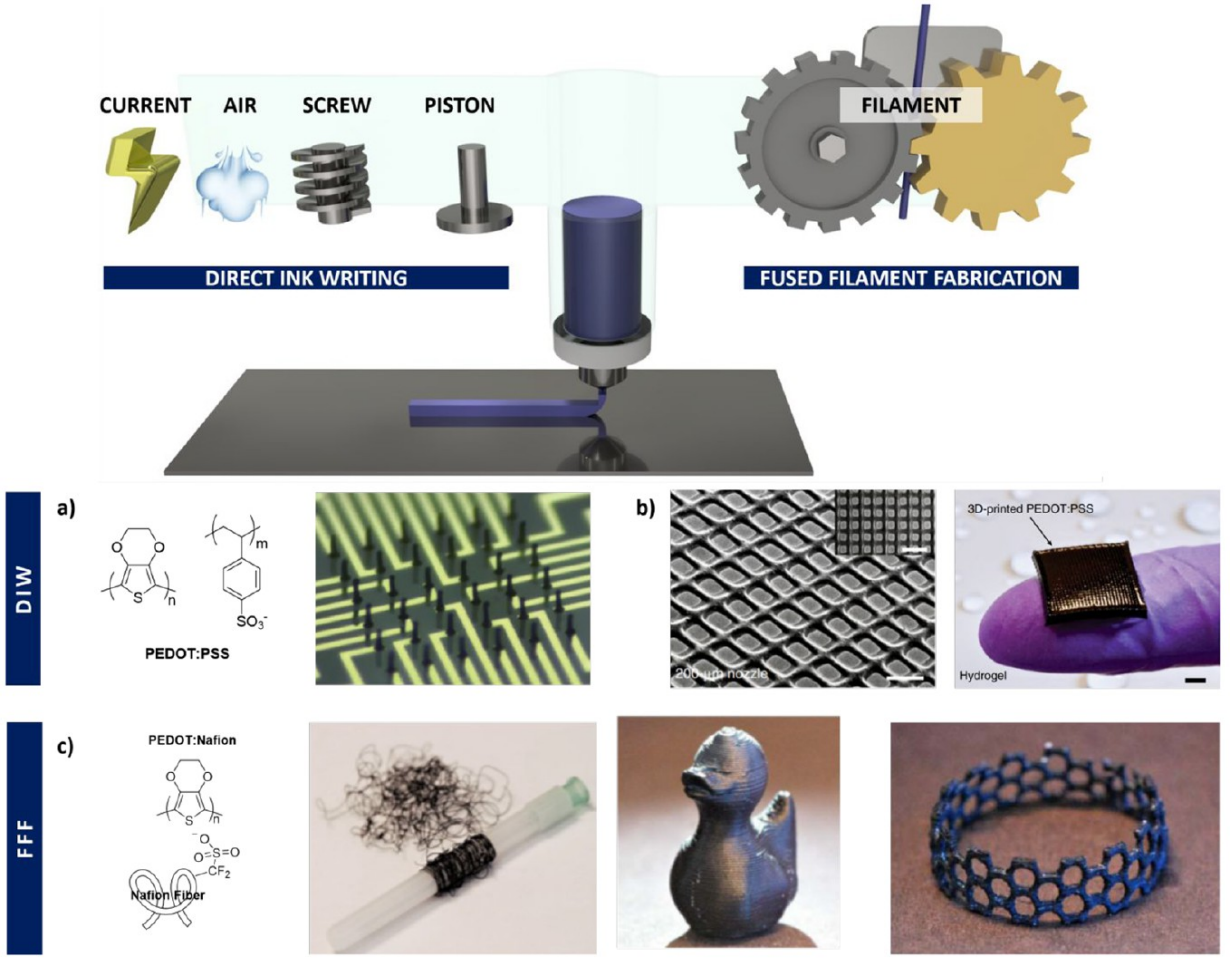
(top) General scheme of an extrusion-based printing method divided in two: DIW, where the material can be driven down through air, screw, or piston, and fused filament fabrication (FFF) where the material is a filament melted by temperature. (bottom) Some examples of printed structures by DIW of PEDOT:PSS: (a) 3D pillar electrodes with 80 μm height and 14 μm diameter. Adapted and reprinted with permission from ref (57). Copyright 2019 Wiley-VCH Verlag GmbH & Co. KGaA. (b) Free-standing 3D layer-by-layer scaffolds with high resolution. Reprinted with permission from ref (58). Copyright 2020 Springer Nature. (c) PEDOT:Nafion forming filament fused for 3D printing. Adapted and reprinted with permission from ref (59). Copyright 2020 American Chemical Society.

Extrusion-based printing consists in the layer-by-layer deposition of a material through a movable nozzle, which follows a specific shape previously programmed using a software. There are two main extrusion printing methods that differ on the way to drive down the polymer. On the one hand, fused deposition modeling (FDM) uses a polymer in the form of a filament moved throughout a gear mechanism straight to a hot end, where the polymer is melted. On the other hand, direct ink writing (DIW) uses polymers that are semimelted, in solutions or pastes, which low down by the action of current, air, pistons, or screws (Figure 2). Besides this, both FDM and DIW methods require the employment of polymers with a specific rheological behavior, viscosity values lower than 10^4^ Pa·s for low shear rates (10^–1^ s^–1^) and 10^1^ Pa·s for high shear rates (10^2^ s^–1^), to be printable as well as to retain the desired shape after printing.^13,51−56^ As mentioned before, pristine conducting polymers do not show the typical rheological behavior of polymers, and they need to be combined with other thermoplastic in the form of blends or copolymers to be able to be processed using extrusion-based printing methods.

#### Poly(3,4-ethylenedioxythiophene)

Very recently, the DIW of PEDOT:PSS has been reported.^57,60^ This new, effective, and versatile PEDOT:PSS processing technique allows a control of the diameter and arrangement of the printed fibers in a precise, localized, and highly efficient manner, thus providing great opportunities for the development of microelectronic array devices (Figure 2a). PEDOT:PSS pillars with different aspect ratios were fabricated by varying the printing parameters, such as the pulling speed, pulling time, polymer solution concentration, and the tip diameter, leading to high-aspect-ratio pillars of 7 μm diameter and 5000 μm height. Furthermore, the employment of an organic solvent—ethylene glycol (EG) or DMSO—and a cross-linking agent—(3-glycidyloxypropyl)-trimethoxysilane (GOPS)—contributes to enhance the water stability of the printed pillars, which is of vital importance if the arrays are used in biological applications. The addition of EG or DMSO into the ink decreases the evaporation rate and stabilizes the printed structure. Regarding the cross-linker, GOPS locks the PEDOT:PSS chains, through the hydrolysis and condensation of silane groups, improving the physical stability of the printed structure upon contact with water. Besides, EG and GOPS also induce an electrochemical stability as assessed by cyclic voltammetry. Furthermore, the printed pillars exhibit high flexibility and robustness. Zhao and co-workers employed the same methodology described before to manufacture controlled shaped three-dimensional layer-by-layer scaffolds using the DIW methodology (Figure 2b).^58^ The resultant scaffold displayed a high Young’s modulus in a dry state (1.5 ± 0.31 GPa), whereas lower values (1.1 ± 0.36 MPa) were achieved in a hydrogel state. The 3D printed structures could achieve high electrical conductivities of 155 S cm^–1^ in a dry state and 28 S cm^–1^ in a wet state, which could be even increased by shear-induced enhancements in the PEDOT:PSS nanofibril alignment by decreasing the nozzle diameter. In addition to this, the scaffolds showed a mechanical and electrical stability, in both dry and hydrogel states, after 10 000 repeated bending cycles. In this line, the addition of triton X to PEDOT:PSS mixed with DMSO provides an excellent viscoelastic behavior with a high mechanical stretchability (above 35% strain) and remarkable self-healing properties with a recovery time lower than 1 s. This self-healing ability was used to fabricate structures by DIW printing, propelling the mixture by action of a piston, which could be used as thermoelectric generators.^61^

Wearable electronic devices have been also developed by combining PEDOT with the well-known semiconducting polymer poly(3-hexyl thiophene) (P3HT). The PEDOT:PSS layer with a thickness of ∼300 nm displayed a low sheet resistance of ∼70 Ω sq^–1^. Then, P3HT:phenyl C_61_ butyric acid methyl ester (PCBM) was used to increase the external quantum efficiency (EQE) of the printed scaffolds, so that P3HT:PCBM printed layers with a concentration of 2.7 mg mL^–1^ led to a thickness of ∼50 nm and increased the EQE up to 25.3%.^62^

Interestingly, alternative PEDOT dispersions and copolymers have been specifically designed for extrusion 3D printing methods. In an interesting approach, PEDOT:PSS was included in the copolymerization of 2-acrylamide-2-methylpropanesulfonic acid and *N*-acryloyl glycinamide (PNAGA–PAMPS) forming stretchable hydrogels with a sol–gel behavior. A flow-like response behavior is observed when the hydrogel is heated to 90 °C, whereas by cooling to 60 °C the solution is solidified. During this sol–gel process the material could be extruded from the needles forming tridimensional shapes.^63^ Another interesting example by Müller et al. is the polymerization of PEDOT with Nafion, to produce melt-spun PEDOT:Nafion fibers, which were used in a fuse filament on 3D printers (Figure 2c). This approach shows an interesting case where PEDOT:Nafion printing structures retained the conductivity, ∼3 S cm^–1^, upon stretching to 100% elongation. Moreover, they demonstrated that 3D printing shapes possessed good performance for organic electrochemical transistors (OECTs).^59^

**Figure 3:**
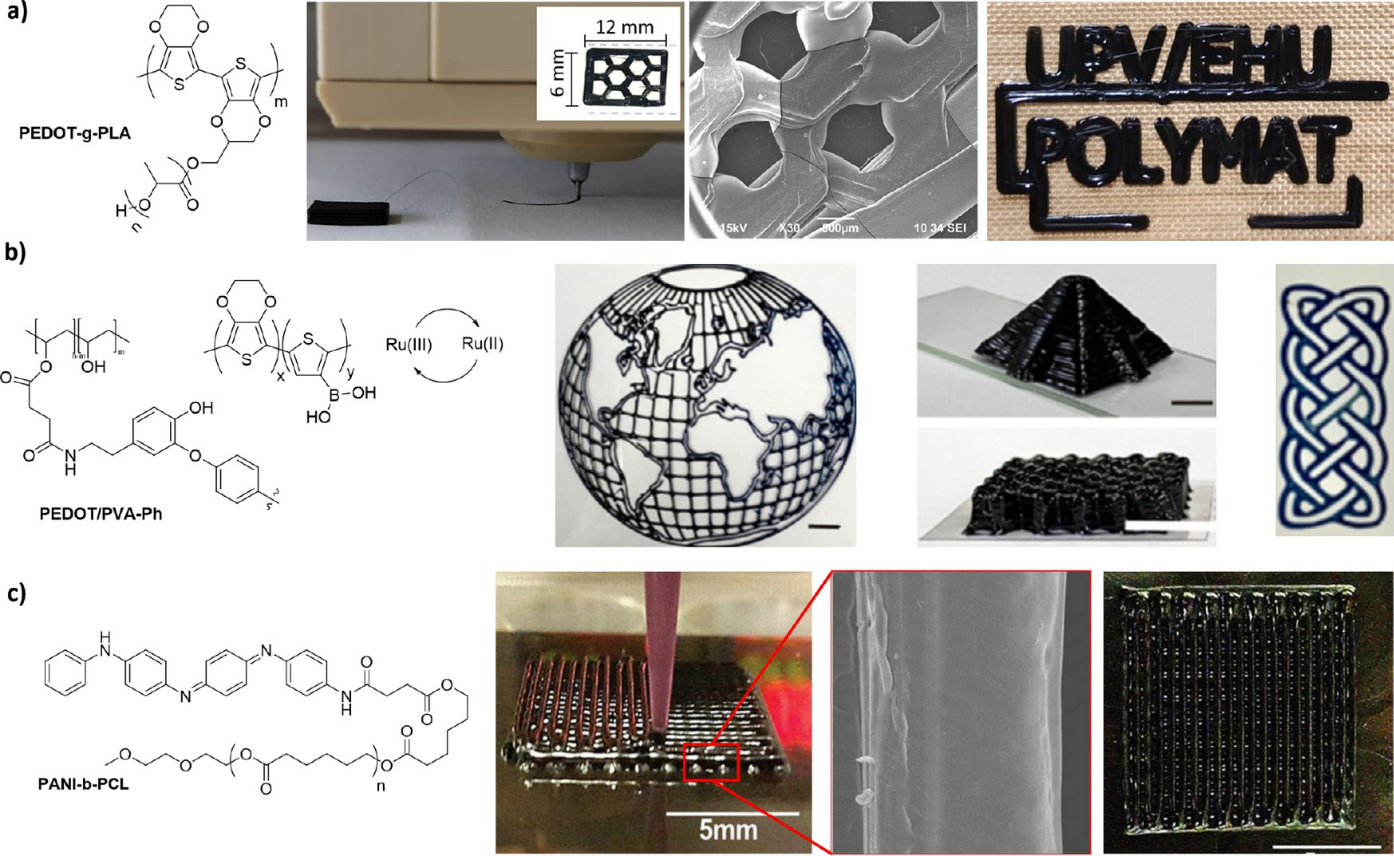
(a) Biocompatible 3D scaffolds based on PEDOT-*g*-PLA for tissue engineering. Adapted and reprinted with permission from ref (64). Copyright 2021 Wiley-VCH GmbH. (b) Printable inks obtained by photopolymerization of PEDOT and coupling reaction of phenols with the catalysis of Ru(II)/APS leading to 3D patterns. Adapted and reprinted with permission from ref (65). Copyright 2021 Springer Nature. (c) Carboxyl-capped tetraaniline graft copolymerized to PCL for DIW method. Adapted and reprinted with permission from ref (66). Copyright 2020 The Royal Society of Chemistry.

Very recently, our group has built up 3D scaffolds by DIW of a conductive graft copolymer, PEDOT-*g*-poly(lactic acid) (PLA), obtained by an oxidative chemical polymerization of PEDOT in the presence of PLA. Interestingly, these scaffolds not only showed excellent biocompatibility properties in contact with cardiomyocytes and fibroblasts but also the formation of tissue-like structures composed of both cell lines (Figure 3a).^64^ An innovative orthogonal photochemistry-assisted printing (OPAP) technique, combining extrusion printing and light-triggered chemistry, has been developed by Yu and co-workers for fabricating three-dimensional tough conductive hydrogels (TCHs) based on PEDOT and tyramine-modified poly(vinyl alcohol) (PVA-Ph). Ruthenium photochemistry was used to trigger two orthogonal photoreactions, a faster phenol-coupling reaction of PVA-Ph (∼27 s) and the polymerization of the conductive polymer precursors including 3,4-ethylenedioxythiophene (EDOT) (∼150 s), leading to a porous PVA hydrogel network with shorter PEDOT chains immobilized in the pores. The inherent properties of these hydrogels, such as stretchability, compressibility, toughness, and conductivity, make them ideal candidates as pressure sensors and temperature-responsive actuators (Figure 3b).^65^

Along the same line, Spencer et al.^67^ developed a biocompatible conductive hydrogel, composed of gelatin methacryloyl (GelMA) and PEDOT:PSS, which was bioprinted to form complex 3D cell-laden structures. First, GelMA/PEDOT:PSS was partially cross-linked by mixing the liquid prepolymer with aqueous CaCl_2_ at 4 °C. Then, the physically cross-linked hydrogel was covalently cross-linked through a photopolymerization of the methacryloyl groups present on the GelMA. The Young’s modulus of these hydrogels varied from 140 to 80 kPa depending on the PEDOT:PSS concentration (0.1 to 0.3 wt %, respectively), whereas the electrical properties were improved with the PEDOT:PSS concentration. Moreover, C2C12 cells were mixed with a selected proportion of GelMA/PEDOT:PSS and printed into the CaCl_2_ bath at 25 °C. In another work, a bioink was obtained by combining methylcellulose and κ-carrageenan (MC/kCA) hydrogels with PEDOT:PSS conducting polymers. The bioink showed a thixotropic behavior that could be tuned by changing the MC/kCA concentration to obtain easy printable bioinks by DIW with a high shape fidelity. In addition, the electrical conductivity increased from ∼1800 to 3000 μS cm^–1^ by increasing the PEDOT:PSS concentration from 0.1 to 0.3 wt %, whereas the impedance decreased. Besides carbohydrates, PEDOT:PSS has been also combined with carbon methyl cellulose (CMC) for Li-ion batteries. PEDOT-CMC electrodes were printed by DIW forming thick electrodes with a high conductivity leading to interconnected tridimensional hierarchical networks, which provide transport paths for Li ions and electrons.^68^ Human embryonic kidney 293 (HEK-293) cells were also incorporated to the bioink formulation, and the 3D printed structures showed a high cell viability (>96%) over a week, resulting in a promising candidate for biomedical applications.^69^

PEDOT nanocomposite materials have been also designed for extrusion 3D printing methods. As an example, Ou and co-workers^70^ incorporated well-dispersed Sb_2_Te_3_ nanoflakes and MWCNTs into a PEDOT:PSS polymer matrix to enhance the thermoelectric performance of the printed hybrid structures. A nominal loading fraction of 85 wt % nanofillers yielded to a high power factor of 41 μW mK^–2^ (S of ∼29 μV K^–1^ and σ of ∼496 S cm^–1^) while maintaining the robustness and mechanical stability of the printed nanocomposites. In another example, Ag-NWs were incorporated within PEDOT:PSS to fabricate transparent and flexible films using roll-to-roll (R2R) and screen-printing technologies.^71,72^ With the application of a potential from 15 to 40 V on the films of PEDOT:PSS/Ag-NWs, a stable temperature from 49 to 99 °C was generated in an interval of 30–50 s leading to a uniform heating and rapid thermal response, whereas the surface temperature of PEDOT:PSS films remained stable compared to the room temperature.^72^ Very recently, Wang and co-workers^73^ have developed hybrid multilayer networks made of inorganic (Ag) and organic (PEDOT:PSS) fibers with 1–3 μm diameters by using inflight fiber printing (iFP), a one-step process that integrates a conducting fiber production and fiber-to-circuit connection. The resulting architecture composed of fiber arrays possessed a high surface area-to-volume ratio, permissiveness, and transparency, which made them ideal candidates to be employed as a cell-interfaced impedimetric sensor, a 3D moisture flow sensor, and noncontact, wearable/portable respiratory sensors.^73^ The difference between Ag and Ag-NWs has been tested by printing multilayer architectures based on PEDOT with the slot-die coating technology.^74,75^

#### Polypyrrole

In the case of PPy processed by extrusion methods, the first example corresponds to the grafting of PPy to the double-bond decorated chitosan (DCh) to form a DCh-PPy copolymer. Subsequently, acrylic acid (AA) was polymerized in the presence of DCh-PPy to form a double network hydrogel composed of poly(acrylic acid) (PAA)/DCh-PPy, which was 3D-printed to fabricate electroconductive scaffolds. The healing properties of the PAA/DCh-PPy hydrogel make possible its elastic modulus recovery (2000 Pa) after breaking down while it passes through the needle. But not only the mechanical properties were recovered; 3D-printed materials showed a 90% electrical recovery in 30 s and 96% in 1 min, which make them excellent candidates to be employed in wearable devices.^76^ As another example, poly(glycerol sebacate), PPy, and nanocellulose were mixed in order to prepare pneumatically impulse inks for DIW printing, building 3D structures used as drug release patches for therapies after a myocardial infarction.^77^ PPy was also combined with alginate to build up biocompatible 3D scaffolds by DIW.^78^ In this line Distler and co-workers have developed three-dimensional porous scaffolds by the DIW of a hydrogel precursor, made of high gelatin-content oxidized alginate-gelatin (ADA-GEL) incorporating PSS and pyrrole (Py), followed by thermal gelation at 22 °C. Subsequently, scaffolds were immersed in an FeCl_3_ solution to oxide Py leading to the formation of the PPy network inside the ADA-GEL matrix and increasing the conductivity (12–16 mS cm^–1^) and stiffness (*G*′ ≈ 1270 Pa) of the hydrogels. These values are in accordance with native cartilage tissue properties, allowing these tissues to be employed as potential 3D scaffolds for electrical-assisted cartilage tissue engineering applications.^79^

In addition to this, Sun and co-workers^80^ found that the morphology of the PPy had an impact on the electrical conductivity of PPy-based scaffolds manufactured by extrusion printing. They employed a printable ink composed of PPy nanostructures (spheres of 50 nm diameter or nanowires of 10 μm length and 100–300 nm diameter) dispersed in a thermosensitive polymer, poly-l-lactide (PLLA), to fabricate scaffolds with ∼100 μm size macropores. Interestingly, it was shown that the electrical conductivity of the 3D scaffolds was higher when PPy was disposed in the form of nanowires into the ink and increased with the PPy concentration. In another work, DIW printing was also employed to manufacture wearable electrodes constituted by alternate layers of a PPy-nanotube ink and a PVA gel ink. It was proven that the 3D printed structure exhibited an excellent mechanical stability where dispersed PPy nanotubes provided a stable channel for ion transport with a 93% retained capacitance at the bending angle of 120°.^81^

Among polypyrrole-based nanocomposites, it is worth mentioning one example of 3D printing PPy with carbon nanotubes. Thus, sensing transducers, emitters, and radio frequency inductors were developed by the uniform dispersion of highly conductive MWCNTs into PPy, which allowed to obtain mechanically suitable inks to be processed by meniscus-guided 3D printing.^82^

#### Polyaniline

PANi has been combined with a biodegradable polyester such as poly(ε-caprolactone) (PCL) by a melt blending of both polymers leading to PANi:PCL inks, which were processed by a screw-assisted extrusion 3D printing to form conducting scaffolds. These scaffolds showed a suitable compressive strength (6.45 MPa), conductivity (2.46 × 10^–4^ S cm^–1^), and human adipose-derived stem cell viability (88%) for bone tissue engineering applications.^83^ In another example, Prasopthum et al.^66^ followed the same strategy, but in this case the conducting polymer ink was obtained by a chemical grafting of PANi and PCL forming a block copolymer, tetraaniline-*b*-PCL-*b*-tetraaniline. 3D scaffolds with a centimeter-scale thickness and interconnected pore nanotextures with nanometre-scale nanofibers were also fabricated for bone tissue applications (Figure 3c). The average diameter of the PCL/PANi nanofibers decreased as the PANi loading increased, whereas the conductivity significantly increased. This can be explained by the fact that the nanofiber can be split off and separated into thinner nanofibers as the conductivity increases resulting in smaller diameters of the PCL/PANi nanofibers.^84^

As an example of PANi nanocomposite inks, reduced graphene oxide (GO) was mixed with PANi to obtain printable PANi/GO inks with shape fidelity, self-sustainability, and electrical conductivity. A 3D printing of this ink gave rise to three-dimensional PANi/GO structures.^85,86^

### 2.3 Electrohydrodynamic Printing

**Figure 4:**
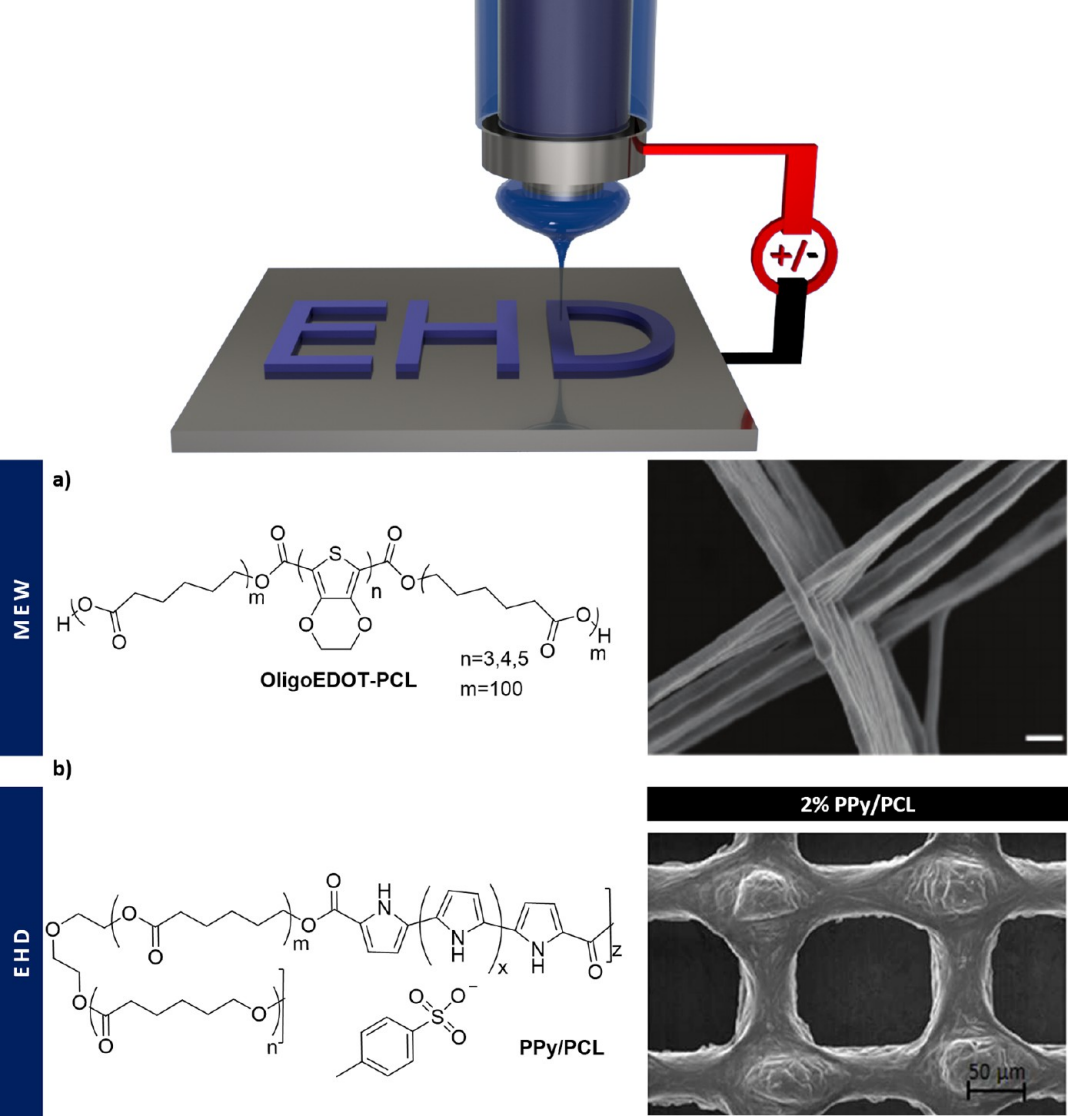
(top) EHD to electrodeposit a polymeric material dissolved in a polarizable liquid through a voltage field. (bottom) Two examples of printed materials using EHD: (a) OligoEDOT-PCL polymer used for melt electro writing forming fibrous scaffolds. Adapted and reprinted from ref (92). Copyright 2020 Molly M. Stevens, Andrea Serio, Ramon Vilar, et al. Published by Wiley-VCH GmbH. (b) PPy-*g*-PCL mixed with PCL leading to an ink that was EHD printed. Adapted and reprinted with permission from ref (89). Copyright 2019 Vijayavenkataraman, Kannan, Cao, Fuh, Sriram, and Lu.

Electrohydrodynamic printing (EHD) is based on the deposition of a material, dissolved in a polarizable liquid, which experiments ion mobility by the action of an electric field that is usually placed between the nozzle and the grounded substrate (Figure 4). The EHD printing method possesses a high resolution and overcomes the limitation related to the nozzle in the inkjet printing methodology. It can be used in a pulsating or jet mode, creating dots or continuous fibers, so the deposition modulates the resolution at the micro or nanoscale domains. The printing quality is affected by the ink properties, such as viscosity, surface tension, electrical conductivity, or dipole moment, besides the process-related factors including the applied voltage, pressure, and flow rate. Overall, at low applied voltages and low ink viscosity a dripping mode is observed at the apex of the Taylor cone. By increasing the voltage and keeping a low ink viscosity at low flow rates, the droplet size is much smaller than the nozzle size giving rise to the microdripping mode. An increase of the flow rate under these later conditions makes the ink eject like a column generating the spindle mode. The employment of high-viscosity inks and high voltages generates a thin liquid jet at the apex of the cone known as cone-jet mode. In this line, by increasing the voltage and flow rate up to very high values, the unstable and uncontrollable multijet mode will be achieved. Among these jetting modes, microdripping and cone-jet provide the required printing process controllability for precision manufacturing.^87^ Moreover, electrodes can be located around the nozzle, which controls the deposition trajectory, achieving a sub-micrometer printing resolution.^88^

#### Poly(3,4-ethylenedioxythiophene)

Wearable electronics have been manufactured by EHD of PEDOT-based inks. That is the case of PEDOT:PSS, which was mixed with poly(ethylene oxide) (PEO) to form a homogeneous EHD printing solution, where the addition of PEO raised the viscosity of the inks. PEO/PEDOT:PSS walls with different layer numbers were printed to obtain three-dimensional structures with wall widths in the range of 49.50–62.50 μm and wall heights from 0.77 to 57.25 μm. By increasing the number of printed layers from 20 to 100, the resistance was significantly reduced from 2.79 ± 0.37 to 0.77 ± 0.05 kΩ cm^–1^, respectively.^90^ In another work, the same authors fabricated multilayer micro/nanofibrous conductive scaffolds through a layer-by-layer printing of this PEO/PEDOT:PSS ink, in the form of nanofibers (470 nm), with a polycaprolactone ink, in the form of microfibers (from 2.5 to 9.5 μm). In each layer, eight PCL microfibers were printed and vertically stacked to form parallel microwalls with a wall spacing of 100 μm. Then, PEO/PEDOT:PSS conductive nanofibers were printed on top with a spacing of 50 μm and a similar orientation to the printed PCL microwalls. This procedure was repeated four times to obtain multiscale conductive scaffolds with a Young’s modulus of 13.1 ± 0.6 MPa that mimic the micro/nanofibrous architectures of the native cardiac extracellular matrix (ECM). The electrical conductivity of the printed PEO/PEDOT:PSS fibers (1.72 × 10^3^ S m^–1^) is much higher than that of the biomaterial blended conductive fibers, at the same time that the impedance of the multiscale conductive scaffolds significantly decreased at physiologically relevant frequencies (<100 Hz) in comparison with pure PCL scaffolds.^91^ Moreover, another example showed the use of end-functionalized oligoEDOT constructs as macroinitiators for the polymerization of PCL forming an electroactive block copolymer. They were used to manufacture fibrous structures, via melt electrospinning writing and solution electrospinning, used for a neuronal culture (Figure 4a). Neurons presented an elongated neurite length under an electrical stimulation demonstrating the promising use of these scaffolds for further tissue engineering applications.^92^ As another example, high-performance organic field-effect transistors (OFETs) and complementary logic circuits were manufactured by EHD. For that purpose, two different polymers were synthesized by the polymerization of PEDOT with PSS-fluoromethyl-derivated PEDOT:[P(SS-*co*-TFPMA)] or PEDOT:PSS with poly(ethylene glycol methyl ether) (PEGME). The printed electrode presented different work function (WF) values, according to the Schottky–Mott rule, to be considered as the next generation of integrated circuits and other multifunctional electronic devices.^93^

#### Polypyrrole

In a recent study, a tailor-made biodegradable and conductive block copolymer, PPy-*b*-PCL, was used as a printable ink to fabricate 3D porous scaffolds by EHD (Figure 4b). Different PPy-*b*-PCL concentrations (0.5, 1, and 2% v/v) were used to obtain scaffolds with average fiber diameters ranging from 33 μm (0.5%) to 44 μm (2%) and an average pore size of 125 μm. PPy-*b*-PCL had a positive effect on the conductivity, which was significantly enhanced from 0.28 mS cm^–1^ (0.5%) to 1.15 mS cm^–1^ (2%), whereas the Young’s modulus slightly decreased from 51 MPa (0.5%) to 35 MPa (2%).^89^

#### Polyaniline

PANi doped with hydrochloric acid (HCl), camphorsulfonic acid (CSA), or a mixture of both led to a formation of conducting polymer inks that could be processed by EHD. The printing of these inks over polymer substrates gave rise to the fabrication of flexible gas sensors.^94^

### 2.4 Light-Based 3D Printing

**Figure 5:**
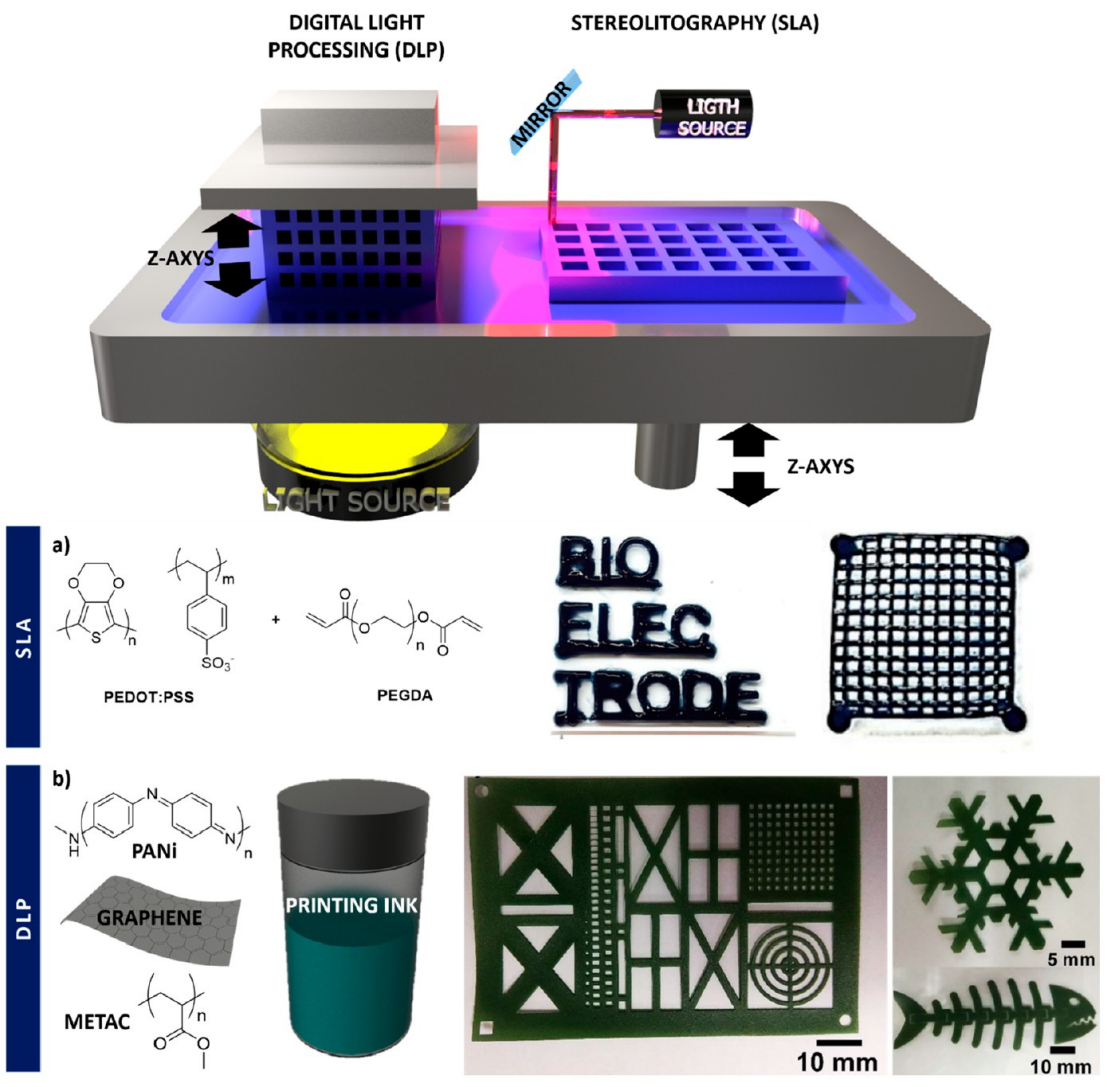
(top) Light-based printing methods divided in two: DLP, where the photopolymerization occurs at the bottom of the vat, and SLA, which uses a laser pulse to polymerize the resin placed on the top. (bottom) Two different examples of light-based printing: (a) PEDOT:PSS mixed with ethylene glycol and PEGDA to form cross-linked structures by the SLA printing method. Adapted and reprinted with permission from ref (97). Copyright 2019 Elsevier B.V. All rights reserved. (b) Composite ink based on PANi, graphene, and METAC to be processed by DLP forming customizable structures with different shapes. Adapted and reprinted with permission from ref (98). Copyright 2020 MDPI.

Light-based 3D printing is based on the photopolymerization of a prepolymer or monomer in a liquid state placed inside a vat through a spatially controlled solidification in a specific shape, forming the 3D structure.^95^ Two main methods are employed, namely, stereolithography (SLA) and digital light processing (DLP) (Figure 5). SLA photocures the resin by a laser beam controlled under a deflection mirror, and the liquid is solidified on the surface where the light spot is scanned. Regarding DLP, a digital micromirror device (DMD) formed by millions of mirrors is used to directly project a 2D image onto the photosensitive material. Moreover, SLA occurs in the top part of the vat, photocuring the resin point-by-point through the laser beam, whereas in the case of DLP the light source projects the entire slice in the bottom part of the vat, where the photopolymerization takes place.^51^ Besides these two methods, selective laser sintering (SLS) can be considered a light-based printing technique, where a photo-cross-linkable prepolymer in a powder is mixed with other polymers, metals, or ceramics to form composites that can be sintered by the action of a high-power laser.^96^ However, CPs are rarely obtained by photopolymerization, which limits the applicability of light-based printing to inks where the CP is dispersed with a light-sensitive curable material.

#### Poly(3,4-ethylenedioxythiophene)

A PEDOT:PSS aqueous dispersion has been printed in situ by SLA into a hydrogel matrix, formed by the photopolymerization of poly(ethylene glycol) diacrylate (PEGDA) (Figure 5a). For that purpose, PEDOT:PSS is formulated in a solution of ethylene glycol and water (1:8). Then, PEGDA containing 0.5 wt % photoinitiator (bis(2,4,6-trimethylbenzoyl)-phenylphosphineoxide) was added to the previous solution to obtain the conducting polymer ink to be printable by SLA. Computer-aided design-based architectural models with square pores and different fiber spacing, 500, 600, and 800 μm, were selected to print the 3D structure by a UV laser exposure leading to well-integrated scaffolds with predesigned geometries. The presence of PEGDA allowed a decrease in the sheet resistance of the 3D printed materials from 968.0 ± 245.1 to 662.0 ± 100.6 Ω sq^–1^. The enhanced electrical properties are attributed to the realignment of a densely packed and highly ordered PEDOT:PSS structure.^97^ Three-dimensional PEGDA:PEDOT structures printed by SLA were also studied by other authors for the long-term monitoring of adsorbed volatile organic compounds. They proposed a proof of concept where variations in the structure and conductivity could be used for monitoring hazardous compounds associated with cumulative adsorption effects.^99^

Regarding PEDOT nanocomposites, as a first example PEDOT:PSS was interpenetrated in situ into nanostructured electrically conductive hydrogels (NECHs) that contained MWCNTs doped with a hydrophilic photoresist, which were ultrafast laser processed as an absorbent 3D scaffold. A two-photon hydrogelation boosted the manufacturing of 3D scaffolds in the nanoscale domain at very high resolution, where the inclusion of PEDOT enhanced the mechanical and electrical properties of 10^–2^ S cm^–1^.^100^ The same methodology was used by Cho and co-workers^101^ to fabricate stretchable transistors operating at less than 1 V. Three-dimensional graphene/PEDOT:PSS structures have been also manufactured by the light-based printing of this hybrid ink over a substrate, making use of a shadow mask with the desired geometry, leading to materials with significant areal capacitances of 23 mF cm^–2^, higher than those obtained by spray-coating (5.4 mF cm^–2^).^102,103^

#### Polyaniline

Graphene sheets mixed with PANi can be embedded in a polyacrylate resin solution to obtain an ink able to be processed by light-based printing. The printed sculptures of the graphene/PANi components showed a low electrical conductivity of 4 × 10^–9^ S cm^–1^, as it was assessed using a four-point probe measurement system (Figure 5b).^104^ As well as polyacrylates, PANi nanofibers can be mixed with polyurethanes and graphene in different compositions to form inks that can be printed using a DLP-type method.^98^

## 3 Trending Applications in (Bio-opto) Electronics and Energy Devices

Most developments related to the 3D printing of conducting polymers are related to applications in the (bio)electronic field as electrodes, sensors, supercapacitors, wearable electronics, electronic skin, human motion sensors, health monitoring, or soft robotics.

### 3.1 Bioelectronic Applications

3D printing represents a powerful tool for building electronic tissue engineering devices due to the possibility of using polymers, which confers to the final structure conductivity, improvement of the mechanical properties, and biocompatibility at the same time that it is able to customize complex architectures to mimic the extracellular matrix and other body tissues or organs to restore damaged body functions.^58^

PEDOT, PPy, and PANi printed structures have shown excellent biocompatibility in contact with cells.^49,80,91^ PEDOT:PSS/PEO conducting fibers incorporated within a PCL matrix guided H9C2 myoblasts and primary cardiomyocytes cell alignments with an enhanced cell proliferation capability.^91^ PPy/PLLA scaffolds showed a cell viability higher than 80% in contact with L929 fibroblasts,^80^ and PPy/PCL scaffolds exhibited an increase of human embryonic stem cells (hESC-NCSCs) proliferation when compared with a pure PCL matrix.^89^ The same behavior was observed in the case of PANi/PCL macrostructures in contact with C2C12 mouse myoblasts^84^ and osteoblast cells.^49^ Besides this, CPs are attractive because of the electrochemical stimulus that can supply to cells in contact with them, which are the primary means of intercellular communication between electroactive cells.^105,106^ In this regard, the stimulation of the substrate is presented as a promising tool for tissue engineering. Microstructured PPy:PVA/collagen scaffolds allowed the electrical stimulation of PC12 cells cultured on them inducing their differentiation, as monitored via type III β-tubulin expression, with extending neurites forming neural networks. The electrical stimulation at precise current values (∼1 mA) induced a significant outgrowth and orientation of neurites compared to unstimulated cells, as assessed by a measurement of the median neurite length with values of 23.34 and 32.71 μm for unstimulated and stimulated cells, respectively.^45,107^

#### Cell-Laden Scaffolds

**Figure 6:**
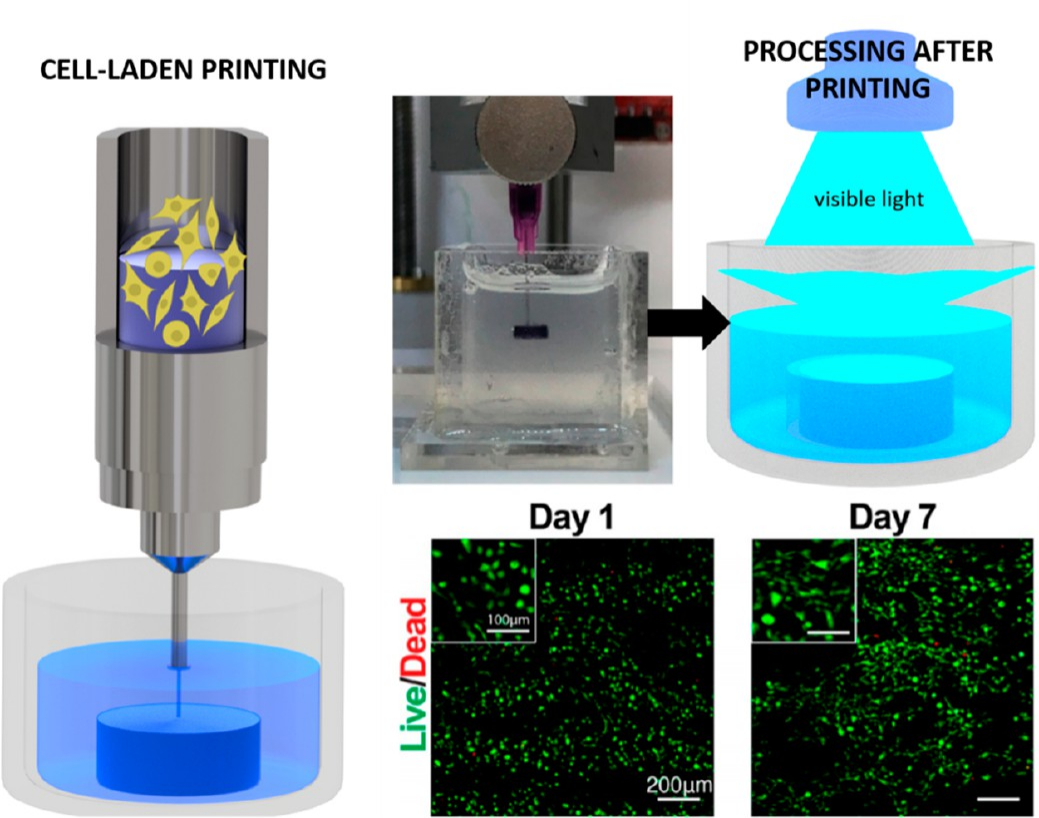
Cell-laden printing of PEDOT:PSS, GelMA, and C2C12 cells within a calcium chloride solution that induced the first physical cross-linking. After bioprinting, the structure is chemically cross-linked across the vinyl group throughout visible light exposure showing an excellent cell viability. Adapted and reprinted from ref (67). Copyright 2019 American Chemical Society.

3D printing also offers the possibility to use functional bioinks with cells encapsulated, commonly known as bioprinting technology, to obtain cell-laden structures.^13,108^ As a first example, human embryonic kidney 293 (HEK-293) cells were encapsulated within MC/kCA/PEDOT:PSS hydrogels leading to a bioink with a controlled electrical conductivity. Bioprinted structures maintained a high HEK-293 cell viability (>96%) over a week, confirming the in vitro biocompatibility.^69^ The same approach has been employed by other authors. Spencer et al.^67^ mixed C2C12 cells with prepolymer printable solutions composed of GelMA/PEDOT:PSS. The quantification of live/dead and actin/DAPI assays showed a high viability and spreading of C2C12 cells in the printed structure, which could be used as electroactive tissue structures on demand from medical scans (Figure 6). As previously, cell-laden conducting polymer scaffolds can be also stimulated electrically to induce cell differentiation. For that purpose, immortalized dorsal root ganglion (DRG) neuronal cells were first encapsulated within a GelMA hydrogel precursor. Further, the cell-laden hydrogel was carefully dispensed in the conductive hydrogel PEDOT:PSS to be light-based printed. The cell-laden conductive hydrogel structure was brought in contact with a neurogenic differentiation medium and subjected to electrical stimulation, 1000 mV per sample of the steady-state direct current (DC) electric field for 2 days. Results showed the neural differentiation of encapsulated DRG cells using various neuronal gene markers, such as brain-derived neurotrophic factor (BDNF), Neurotrophin-3 (NT-3), and erbB2.^97^ Another example was shown by Travas-Sejdic et al.^57^ who encapsulated human neural stem cells (NSCs) within a conductive polysaccharide-based biogel to be printed onto conductive PEDOT:PSS pillars. Gel-laden cells differentiated into neurons and glia with or without stimulation; however, stimulated constructs comprised large numbers of densely packed cells abutting the underlying substrate, with polarized neuronal cells exhibiting axons and dendritic arborizations, with respect to unstimulated ones. These results allow an anticipation of the potential utility of this platform for electroceuticals, drug screening, and regenerative medicine.

#### Biosensors and Electrophysiology

**Figure 7:**
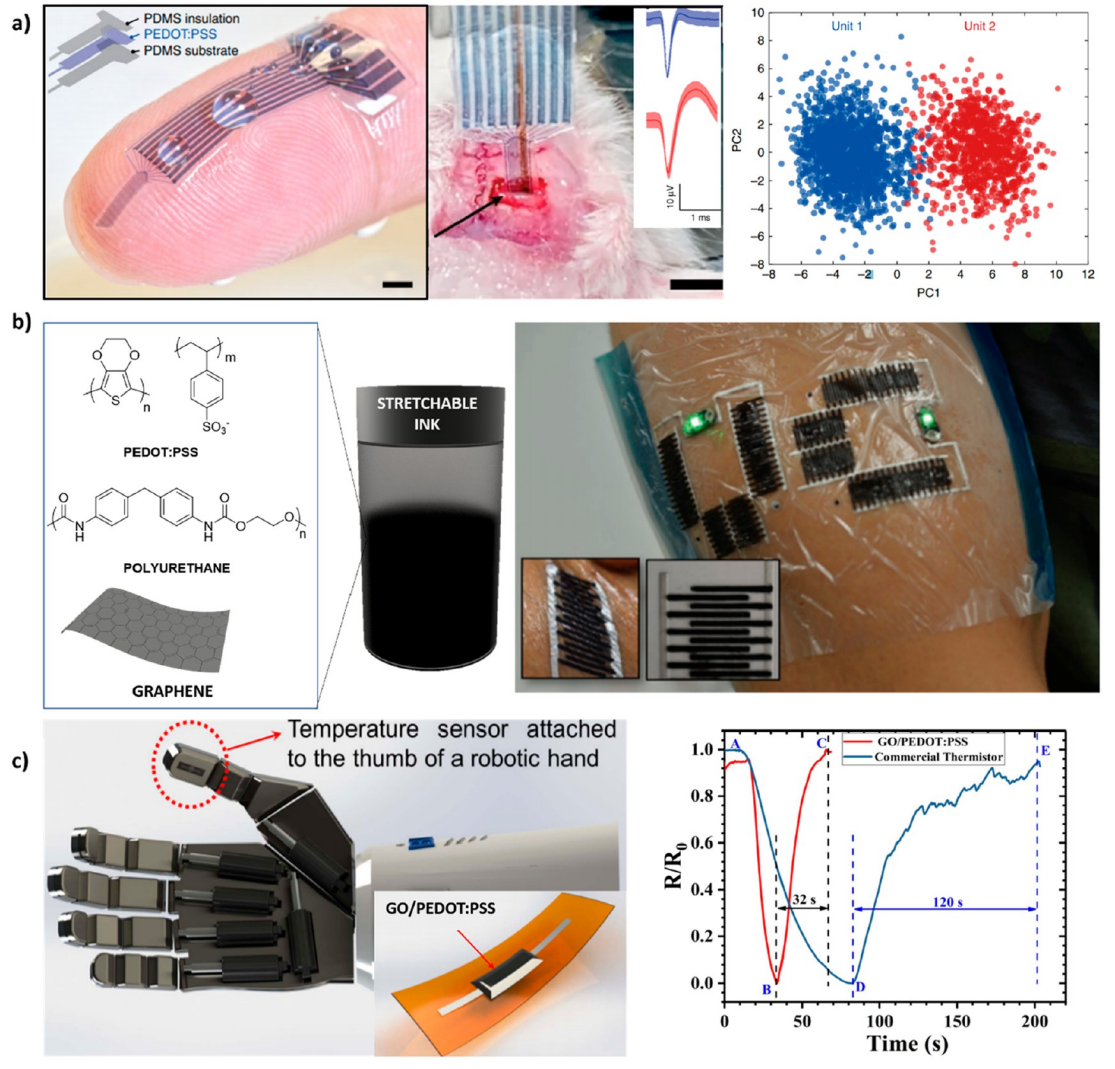
Different setups manufactured by 3D printing of CPs to be employed as sensors. (a) PEDOT:PSS electrodes printed with high resolution by extrusion-based printing and implanted in the mice cranium for electrophysiological recordings and average two units spike waveforms recorded from individual channel of the probe over time. Reprinted with permission from ref (58). Copyright 2020 Springer Nature. (b) Polyurethane/graphene/PEDOT:PSS ink used for manufacturing ultrathin devices on a flexible substrate to be attached on body parts without losing capacitance. Adapted and reprinted with permission from ref (103). Copyright 2019 Wiley-VCH Verlag GmbH & Co. KGaA. (c) Temperature sensor based on PEDOT:PSS and GO inserted in a robot interface allowing the detection of physical interactions with real-world objects, improving a commercially available thermistor. Adapted and reprinted with permission from ref (41). Copyright 2020 IEEE Sens under Creative Commons License (http://creativecommons.org/licenses/by-nc-nd/3.0/).

As a representative example, a PEDOT:PSS-based electrode was manufacured by inkjet printing to record physiological data.^29^ This wearable device was tested by recording electrocardiograms (ECGs) from a volunteer. Measurements were conducted between the two forearms, over a period of 40 days at different time intervals (*t* = 0, 4 h, 8 h, 24 h, and 40 d). The mean signal-to-noise ratio (SNR), calculated at *t* = 0, was 12.93 ± 0.80 dB and remained constant during the first 24 h of recording. After 40 days, the printed electrode exhibited signs of slight degradation with a decrease in SNR to 7.28 ± 5.28 dB. Furthermore, a cholinium lactate-based ionic liquid gel ink was chosen to be printed on top of PEDOT:PSS layers to improve the contact between the conducting polymer and skin, as such gels lead to high-quality contacts with a long-term stability. These gel-assisted electrodes made low impedance contacts to the skin and yielded recordings with a quality comparable to commercial wet Ag/AgCl electrodes but with the advantage that there were not dried and used a more compatible format with wearable diagnostics. These results allow a validation of the use of the designed textile electrodes as customizable health monitoring devices for electrophysiology recordings. 3D printing PEDOT:PSS microstructures can be also employed as soft neural probes for an in vivo single-unit recording.^58^ In that case, in vivo electrophysiology was performed in young adult mice after a probe implantation in the mice cranium by surgery. An electrophysiological recording was performed by coupling the 3D-printed soft neural probe with Neuro Nano Strip Connectors, and the results showed that the 3D-printed soft neural probe could record continuous neural activities in a freely moving mouse over two weeks (Figure 7a).

Very recently, Wang et al.^73^ designed noncontact, wearable/portable respiratory moisture sensors based on a 3D printing of Ag and PEDOT:PSS fibers. In the first configuration, a single-layer PEDOT:PSS fiber array is printed on a plastic frame to be subsequently attached to the exterior of a disposable mask for respiration rate monitoring. This sensor showed good responsive resistance of the fiber array returned to the baseline level in less than 3 s after a normal breathing, which is very important for fast breathing detection. In the second configuration, a trilayer 3D sensor was fabricated by sandwiching a poly(vinylidene fluoride-*co*-trifluoroethylene) [P(VDF-TrFE)] midlayer, able to detect sound by acoustically driven piezoelectricity, within the PEDOT:PSS fiber arrays. This sensor can be attached to a phone camera for a simultaneous collection of image, sound, and breath humidity content variations, to detect the respiratory moisture flow that permeates from a mask. An additional advantage of this sensor is that it allows an identification of the signature of breath leakage distinguishing between breathing and coughing. These findings are particularly important in the global outbreak of acute respiratory diseases, that is, coronaviruses, rhinoviruses, etc., to mitigate transmission risk.^73^

#### Electronic Skin and Robotics for Tissue Engineering

Further applications of 3D printing technology in a tissue engineering field comprise the fabrication of electronic skin (e-skin) materials, which are able to mimic the properties of some human organs to be employed in regenerative medicine.^109^ Wearable human-interactive devices able to enhance the comfort, convenience, and security of humans are attractive for use in this field covering a wide range of applications, from robotics to clinical monitoring.^110^ It is worth noting that, in order to develop e-skin materials that enable a seamless integration with the human body, a combination of different properties, such as stretchability, self-healing ability, high mechanical toughness, tactile sensing capability, and stimuli responsiveness, is required.^111−113^

As a first example, a super tough electro-tendon robotic finger able to transmit an actuation force in robotic hands has been manufactured by the printing of a hybrid ink composed of spider silk, PEDOT:PSS, and single-walled carbon nanotubes (SWCNTs). Spider silk provides a toughness to the tendon-driven transmission system, whereas a mechanical flexibility and electrical conductivity are given by PEDOT:PSS. Furthermore, the incorporation of SWCNTs into the silk contributes to an overall reinforcement of all these properties. The electron-tendon had a toughness of 420 MJ m^–3^ and a conductivity of 1.077 S cm^–1^, which was maintained after 40 000 bending stretching cycles due to the fact that the wrinkled structure flattened upon stretching, preventing any changes in the conductive path. In addition to this, it was able to transmit signals and force from the sensing and actuating systems simultaneously, to be used to replace the single functional tendon in a humanoid robotic hand to perform grasping functions.^114^ As another example, stretchable microsupercapacitors (MSCs) were developed by a laser-induced printing of polyurethane, graphene, and PEDOT:PSS. This MSC could be attached to a human finger or other body parts, demonstrating an excellent flexibility and compatibility with artificially intelligent devices that undergo bending and stretching. It was mechanically stable after 1000 bending cycles showing high capacitance retention (∼98%) and only a 1% capacitance loss, compared with the flat state, when subjected to random twisting and folding mimicking the conditions of wearing the device on a human finger (Figure 7b).^103^ Apart from those works, PANi-based sensors with a high stretchability (∼500%) and electrical conductivity (0.12 S cm^–1^) were fabricated by a screen printing of this conducting polymer in combination with poly(acrylic acid) (PAA) and phytic acid. While stiffness is given by PANi, the presence of PAA as a soft counterpart allowed to form intermolecular hydrogen bonds with PANI chains along with electrostatic interactions leading to self-healing materials able to mimic the dynamic network structure of the dermis. Moreover, the presence of phytic acid as dopant allowed to create additional physical cross-linking points giving rise to a 600-fold increase of the electrical conductivity and a fourfold strength increase.^115^

Temperature sensing is another important parameter that must be measured during the physical interaction of robots with real-world objects for e-skin applications. For that purpose, Vuorinen et al.^40^ fabricated skin-conformable temperature sensors by inkjet printing of a graphene/PEDOT:PSS ink on top of a skin-conformable polyurethane plaster (adhesive bandage).^40^ The initial resistance of the material before cycling measurements was ∼9 kΩ. Then, samples were heated from 35 to 45 °C to be subsequently cooled back down to 35 °C, mimicking the human skin temperature and possible temperature deviations on top of the human epidermis. It was pointed out that the resistance decreased when the temperature increased; thus, graphene/PEDOT:PSS behaves as a negative temperature coefficient (NTC) material. The average temperature coefficient of resistance (TCR or α) of the sensor was 0.047% per degree Celsius. In this regard, another skin-conformable printed temperature sensor was fabricated with PEDOT:PSS incorporating GO as a temperature-sensitive layer and silver (Ag) as a contact electrode with a sensitivity of 1% per degree Celsius. By increasing the temperature from 25 to 100 °C, an 80% decrease in resistance across the GO/PEDOT:PSS layer is measured. The sensor’s response is stable and repeatable under static and dynamic bending (for 1000 cycles) conditions. This sensor was even attached to a robotic hand to allow the robot to act by using temperature stimuli (Figure 7c).^41^

### 3.2 Energy Devices

CPs processed by 3D printing have been also employed in other fields to act as wearable storage devices, supercapacitors, transistors, and photodetectors, among others.

#### Wearable Energy Storage Devices

Organic electrochemical transistors (OECTs) have been manufactured by the 3D printing of PEDOT:Nafion fibers and being able to retain their conductivity (3 S cm^–1^) upon stretching to 100% elongation.^59^ In another case, OFETs were fabricated by the EHD of PEDOT:PSS showing excellent electrical properties, including the on/off switching ratio higher than 10^7^ and the highest carrier mobility greater than 1 cm^2^ V^–1^ s^–1^.^93^ Another example is based on the manufacture of stretchable transistors by an inkjet printing of PVDF and PEDOT:PSS together with SWCNT, which displayed mobilities of 30 cm^2^ V^–1^ s^–1^ and currents per channel width of 0.2 mA cm^–1^ at a 1 V operation voltage.^116^

Besides this, stretchable and self-healing wearable thermoelectric generators (TEGs) were fabricated by the 3D printing of a ternary composite of PEDOT:PSS blended with a polymeric surfactant, Triton X-100 as a healing agent, and DMSO as a thermoelectric performance booster. The TEGs exhibited a power output of 12.2 nW, of which more than 85% was retained after damage induced by a repetitive cutting.^61^

Graphene-doped PEDOT:PSS 3D printed structures can be also employed as MSCs with a significant areal capacitance of 5.4 mF cm^–2^ and good capacitance retention ∼75% (from 10 to 1000 mV s^–1^).^102^ Another example is the DIW of PPy leading to high-performance supercapacitors with high areal capacitance (200 mF cm^–2^) able to be 93% retained after bending.^81^ Flexible MSCs based on PANi have been also fabricated by DIW of this conducting polymer together with graphene oxide reaching areal capacitances in the range of 153.6–1329 mF cm^–2^.^85,86,117^

Electrodes of PEDOT:PSS combined with CMC were printed by DIW and used for Li-ion batteries. The PEDOT:PSS/LiFePO_4_ electrode exhibits a high areal capacity (5.63 mAh cm^–2^) and high stability after 100 cycles, maintaining 92% of its capacitance. Cui and co-workers indicated that the tortuosity or square pores geometry in the micrometer size provided effective transport paths for Li^+^ and electrons, which is beneficial for the electrolyte penetration and charge transfer. However, an extra-thick electrode (up to 1.43 mm) hindered the transmission dynamics and decreased the rate capability.^68^

#### Sensors, Photodetectors, Light-Emitting Cells, and Solar Cells

Regarding sensors, PEDOT has been the main conducting polymer processed for this application. Temperature sensors based on PEDOT:PSS and incorporating CNT or carbon were able to reach TCR as high as 0.25% per degree Celsius and 0.61% per degree Celsius, respectively.^110,118^ Humidity sensors based on PEDOT:PSS doped with GO were also developed. The results showed that the sensor exhibited excellent humidity sensing properties in a wide response range (0.13–68.46%), short response and recovery times (39 and 57 s, respectively) as well as a high repeatability and flexibility with no significant variation of the sensor resistance after folding 200 times.^119^ On the basis of the same principle, film heaters were manufactured by screen printing of PEDOT:PSS and Ag-NWs. The composite film with 74.1% transmittance could be heated from 41 to 99 °C at the driven voltages from 15 to 40 V, showing a uniform heating and rapid thermal response.^72^

Organic photodetectors were also developed by the multilayer inkjet printing of PEDOT:PSS in combination with P3HT:PCBM. The printed photodetector exhibited a photoresponse when photoexcited at 405, 465, 525, and 635 nm, showing the highest responsivity at 405 nm and a strong frequency dependence from 25 to 1000 Hz.^120^ Another trending application of PEDOT:PSS-based printed materials is focused on the fabrication of the highly demanded light emitting cells (LECs). The inkjet printing of PEDOT:PSS together with a polymer electrolyte based on a PCL-*co*-TMC:TBABOB allowed to manufacture LECs with a luminance over 10^4^ cd m^–2^ and efficiencies of 2 cd A^–1^.^30^ Furthermore, the printing of these materials over ultraflexible parylene C substrates, usable for conformable electronics, allowed the obtainment of wearable devices with a maximum brightness of 918 cd m^–2^ and stable operation at a luminance higher than 100 cd m^–2^ for 8.8 h, with a turn-on time of 40 s to reach 100 cd m^–2^.^31^

Electrodes manufactured by the 3D printing of conducting polymers have found applications in the fabrication of solar cells. That is the case of fully inkjet printed multilayer Ag-NWs and PEDOT:PSS electrodes, where Ag-NWs are placed at the bottom and top, leading to semitransparent organic solar cells with a power conversion efficiency of 4.3%/cm^2^.^42^ As another example, organic solar cells with a total area of 186 cm^2^ were manufactured by a multilayer printing of PEDOT:PSS and P3HT:PCBM with Ag placed at the bottom and top, as previously. These cells showed an active-area power conversion efficiency of 1.6% with a good operational stability both under low light and 1 sun conditions.^74^

## 4 Conclusions and Future Perspectives

This article aims at presenting a comprehensive overview concerning the additive manufacturing of most common conducting polymers, such as PEDOT, PPy, and PANI, employing 3D printing techniques. The additive manufacturing of CPs presents great opportunities in the design of new devices and applications in the (bio)electronic field. However, CPs show important limitations in terms of processability. Therefore, their combination with other polymers and nanoadditives that improve their processability at the same time that keeps the high conductivity is needed for their processing by additive manufacturing methods. In this regard, the 3D printing of CPs has started to be explored very recently and portrays a field with a huge number of possibilities and applications in the future. A summary of all works referenced in this review article is collected in Table 1, where conducting polymers mixed with other polymers and/or conducting fillers are listed and related with the 3D printing technique employed, the electrical properties, and final applications.

**Table 1:** Main Conducting Polymers Processed Employing Different 3D Printing Technologies and Relation of the Printed Materials with the Electrical Properties and Final Applications

conducting polymer	secondary polymer	conducting filler	3D printing technique	electrical properties	final structure dimension/applications	ref
PEDOT:PSS			inkjet	0.8 kΩ cm^–2^	2D-3D/electrophysiology	(29)
				0.2–1.8 cd A^–1^, 20 mA cm^–2^	LECs, wearable electronics	(31)
			DIW	15–50 S cm^–1^	3D/sensors and soft electrochemical probes	(60)
				28–155 S cm^–1^	2D-3D/soft neural probes, wearable electronics	(14,58)
				137 S cm^–1^	3D/thermoelectric generators	(61)
	PCL-*co*-TMC:TBABOB		inkjet	1.2 cd A^–1^, 0.3 lm W^1–^	3D/LECs	(30)
	EMIM:ES		inkjet	900 S cm^–1^	3D/biolectronics devices	(15)
	P3HT:PCBM		inkjet	0.0019 A W^1–^	2D/photodetector	(120)
	P3HT:PCBM	Ag-NWs and ZnO	R2R		2D/solar cells	(75)
	PEGDA		SLA	662–968 Ω sq^–1^	3D/neural tissue engineering	(97)
				0.055 S cm^–1^	3D/volatile organic compounds’ adsorbents (VOCs)	(99)
	PEO		EHD	0.8–2.8 kΩ cm^–1^	3D/wearable electronics	(90)
	PCL		EHD	1.72 × 10^3^ S m^–1^	3D/cardiac tissue engineering	(91)
	PEGME P(SS-*co*-TFPMA)		EHD	425–450 S cm^–1^	2D-3D/organic field-effect transistors (OFETs)	(93)
	PNAGA:PAMPS		DIW	0.2–2.2 S m^–1^	3D/biosensors and electroactive scaffolds	(63)
	GelMA		DIW		3D/cell-laden structures	(67)
	MC/kCA		DIW	1800–3000 μS cm^–1^	3D/cell-laden structures	(69)
	cellulose:alginate		DIW	5.7 S m^–1^	3D/energy storage	(121)
	carboxymethylcellulose		DIW	10.3 S cm^–1^–0.9 S cm^–1^	3D/energy storage	(68)
	PVA-Ph		DIW, light based	0.5–3.5 S m^–1^	3D/sensors, actuators	(65)
		CNT	Screen/shadow mask printing	63 mΩ sq^–1^	3D/wearable sensors	(115)
	spider silk	SWCNT		1077 S cm^–1^	3D/electron-tendon	(114)
	PVDF		inkjet	0.2 mA cm^–2^, 30 cm^2^ V^–1^s^–1^	2D/Stretchable transistors	(116)
		MWCNT	inkjet	6.7 S cm^–1^	2D/-	(39)
			aerosol-jet	41 μW/mK^2^, 29 μV K^–1^, 496 S cm^–1^	2D/Energy storage wearable devices	(70)
			light-based	0,4 S cm-^1^	3D/nanorobotics	(100)
		graphene	inkjet	9 kΩ	2D/electrophysiology	(40)
					2D/slectronic skin (eSkin)	(41)
		graphene	aerosol-jet	1080 μF cm^–2^	2D/wearable power supplies	(102)
			light-based	5.4–23 mF cm^–2^	2D/wearable electronics	(103)
		Ag-NWs	inkjet	10^2^ mA cm^–2^	3D/organic solar cells	(42)
		Ag-NWs	R2R	0.5–1.3 Ω sq^–1^	2D/Electrodes	(71,72)
		Ag	iFP	70 S cm^–1^	3D/cell-interfaced impedimetric sensor, moisture flow sensor, and noncontact, wearable/portable respiratory sensors	(73)
		Ag and ZnO	R2R	1000 W m^–2^	2D/solar cells	(74)
PEDOT	Nafion		DIW	3 S cm^–1^	3D/electrochemical transistors	(59)
	PLA		DIW	1.8–300 μS cm^–1^	3D scaffolds/tissue engineering	(64)
PPy			inkjet	0.7 S cm^–1^	2D/eelectronic devices, tissue engineering scaffolds	(44)
	collagen		inkjet	1.1 S cm^–1^	2D/neural tissue engineering	(45,107)
	PCL		EDH	0.28–1.15 mS cm^–1^	3D/peripheral neuronal regeneration	(89)
	DCh, PAA		DIW	25–70 S cm^–1^	3D/human motion detection	(76)
	PLLA		DIW	0.48 S cm^–1^	3D/electroactive tissue engineering scaffolds	(80)
	PVA		DIW	200 mF cm^–2^	3D/supercapacitors, wearable storage devices	(81)
	poly(glycerol sebacate):cellulose		DIW	34 mS cm^–1^	3D/cardiac patches	(77)
	alginate		DIW	4.07–6.33 mS cm^–1^	3D/tissue engineering	(78)
	alginate-gelatin		DIW	12–16 mS cm^–1^	3D/cartilage tissue engineering	(79)
		MWCNT	meniscus-guided 3D printing	25 S cm^–1^	3D/sensing transducers, emitters, and radio frequency inductors	(82)
PANi			inkjet aerosol	480 F g^–1^	3D/supercapacitors, batteries, biosensors, bioelectrodes	(48)
			EHD	76–755 kΩ	3D/sensors	(94)
	PCL, gelatin, SG5		inkjet	10^–3^ S cm^–1^	3D/bone tissue engineering	(49)
	PCL		DIW	0.25 × 10^–4^ S cm^–1^	3D/bone tissue engineering	(83)
				6.2 × 10^–6^ S cm^–1^	3D/cartilage tissue regeneration	(66)
					3D/tissue engineering	(84)
		graphene	DIW	150–1300 mF cm^–2^	3D/flexible microsupercapacitors	(85,86)
				238 F g^–1^	3D/electrodes	(117)
	polyacrylate	graphene	light-based, spray	4 × 10^–9^ S cm^–1^	3D/bioelectronics devices	(104)
	polyurethane	graphene	DLP	1.37 × 10^–6^ S cm^–1^	3D/biomedical devices	(98)
		Ag-NWs	inkjet	50 Ω sq^–1^	2D/electrodes	(50)

Among different CPs used for 3D printing, PEDOT is the most studied, especially the commercial dispersion PEDOT:PSS, which has resulted in the preferred material for different authors. Furthermore, PEDOT-based inks have been processed employing several 3D printing techniques, that is, inkjet, extrusion, electrohydrodynamic, and light-based printing. But not only that, PEDOT:PSS has been also processed in combination with other polymers, solvents, hydrogels, and conducting fillers leading to tailor-made inks with tunable conducting properties to be employed in a wide range of applications. Other conducting polymers, such as PPy and PANi, have introduced a high degree of novelty and have been used for the synthesis of tailor-made conducting copolymers. In fact, very elegant tetramers or block copolymers based on PPy and PANi have been used for direct ink writing, electrohydrodynamic, and inkjet printing, leading to three-dimensional conducting scaffolds.

However, P3HT, which is one of the most popular semiconducting polymers used for solar cell applications, has been scarcely investigated using additive manufacturing. There are only a few examples concerning the additive manufacturing of P3HT:PCBM inks, employing SLA,^122^ slot–die coating,^123^ and aerosol-jet printing,^101,124^ leading to artificial human eyes, medical sensors, generators, and transistors, respectively. In our opinion the AM of this polymer should find many opportunities in the near future. Similarly, it is worth mentioning that other (semi)conducting polymers such as poly(*p*-phenylene-vinylene) (PPV), polyfuran (PF), and polythiophenes (PTh) have not been explored yet, which shows many future opportunities.

Moreover, we believe that nanocomposites formed by conducting fillers and CPs and processed by 3D printing methods have been under-investigated. Besides works referenced previously in this review, there is only an example where PPy is mixed with black phosphorus to be printed by the extrusion-based method leading to electrodes for energy storage.^125^ However, other bidimensional materials, that is, inorganic MXenes, metal–organic frameworks (MOFs), and graphenes, among other emerging 2D nanomaterials, represent a big challenge to be mixed with CPs and processed by 3D printing to form hybrid conducting materials with several possibilities and new applications. In addition to this, there are emerging printing methodologies, such as selective laser sintering, two-photon polymerization printing (2PP), volumetric printing, and fused filament fabrication, that still remain mostly unexplored for conducting polymers opening a new door of possibilities and applications in the future. Finally, it is worthwhile to note that ionically conductive matrices, such as ion gels, could be also processed employing all these 3D printing techniques and employed for the applications previously described.^126^
